# Medical Society of the County of Erie

**Published:** 1903-05

**Authors:** Chauncey P. Smith


### Medical Society of the County of Erie.

*Special Meeting, held March 18, 1903.*

Reported by CHAUNCEY P. SMITH, M. D., Acting Secretary.

The President, Dr. Ernest Wende, took the chair at 4.15 o'clock p.m.

Dr. H. R. Hopkins moved that Chauncey P. Smith be chosen acting secretary. Carried.

The president called upon Dr. H. R. Hopkins to state the object of the meeting, who said that it was to give support to a legislative measure which was the result of a great deal of experience and study—namely, a bill to do away with the dual system of education in the State of New York. It was well known that the remarkably efficient institution, the University of the State of New York, conducted by the regents, had been in existence for over one hundred years. There was also a department of education under a superintendent of education, who had charge of the common schools. It is the belief of those who have examined the matter that it is best to unify and hold one body responsible for education. In other words, the object is to eliminate politics from our school system, and to substitute experienced educators as the governing body.

Dr. C. Diehl moved that the chair appoint a committee of five to express fully to the legislature the ideas of the medical profession in regard to the subject under consideration. Carried. The president appointed as such committee Drs. Diehl, Hopkins, W. W. Potter, Abbott and Bull who reported the following preamble and resolutions:

Whereas, The Medical Society of the County of Erie, State of New York, has called this meeting of the members of the medical profession of the city of Buffalo and the County of Erie; and,

Whereas, As physicians and representatives of our several societies, we are unreservedly and unequivocally committed to the right of individual opinion and also to the paramount necessity for unity in action; and,

Whereas, We have for years noted with regret the lack of unity in the educational systems of our state; and,

Whereas, Legislation is now under consideration for the purpose of bringing about a much needed unification; and,

Whereas, We believe that education is the foundation of the protection and promotion of the public health, the safeguard of civil and religious liberty, the bulwark of civilization, and that in education only the most intelligent methods should prevail, also that in education there should be unity of direction; and,

Whereas, The University of the State of New York was conceived in the highest wisdom, and for more than a century has been conducted with marvelous ability and success and now holds the universal confidence of the educators and the educated; and,

Whereas, It is the purpose of a bill now before the legislature at Albany, know as the Stevens bill, to provide for such unification of the educational system of the State under the direction of the regents of the University; therefore

*Resolved*, That the medical profession of the City of Buffalo and the County of Erie hereby declares itself heartily in favor of the Stevens bill; that we urge our representatives to use all honorable efforts to secure its passage; that a copy of this declaration be sent to each member of our delegation and a copy to the Honorable James Russell Parsons, Jr., Secretary of the University, Regents' office, Albany, N.Y.

(Signed) CONRAD DIEHL,
ALEX. T. BULL,
WILLIAM WARREN POTTER,
HENRY R. HOPKINS,
GEORGE ABBOTT,
*Committee.*

The report was then debated by William Warren Potter, Eli H. Long, George Abbott, of Hamburg; A. W. Bayliss, W. B. Jolls, of Orchard Park; William C. Krauss and Chauncey P. Smith, after which it was unanimously adopted.

The legislative committee was charged with carrying out the provision to notify the Erie County delegation in the legislature, after which the meeting adjourned.

---

THE TREATMENT OF CHRONIC CYSTITIS.—Cases of chronic cystitis are generally of infective origin, many resulting from the upward extension of an inflammatory process in the posterior urethra. A particularly distressing form of the disease is that accompanying enlargement of the prostate. While in the treatment of chronic vesical catarrhs, local measures are often required, much benefit is derived from the internal administration of urinary antiseptics and antiblennorrhagics.

A new preparation embodying all the advantageous features of this class of remedies is uriform. Its antiseptic power is due to the liberation of formaldehyde in the urinary tract, thus preventing ammoniacal fermentation and keeping the urine in a sterile state.

Associated with this is the well-known beneficial action of saw-palmetto upon the mucous membrane of the bladder, as manifested by a diminution in the secretion of mucus and relief of the tenesmus. The other constituents of uriform (damiana and nux-vomica) exert a general tonic effect upon the nervous system, and help to overcome the relaxed condition of the mucous membranes usually present.

Uriform is palatable and well adapted for continued administration in these chronic cases.

